# Risk Factors for Influenza A(H1N1)pdm09 among Students, Beijing, China

**DOI:** 10.3201/eid1902.120628

**Published:** 2013-02

**Authors:** Yang Zheng, Wei Duan, Peng Yang, Yi Zhang, Xiaoli Wang, Li Zhang, Surabhi S. Liyanage, Quanyi Wang

**Affiliations:** Author affiliations: Beijing Center for Disease Control and Prevention, Beijing, People’s Republic of China (Y. Zheng, W. Duan, P. Yang, Y. Zhang, X. Wang, L. Zhang, Q. Wang);; University of New South Wales, Sydney, New South Wales, Australia (S.S. Liyanage)

**Keywords:** pandemic, influenza, viruses, influenza A(H1N1)pdm09 virus, influenza A(H1N1)pdm09, risk factors, students, Beijing, China

## Abstract

To identify risk factors associated with influenza A(H1N1)pdm09 among students in Beijing, China, we conducted a case–control study. Participants (304 case-patients and 608 controls, age range 6–19 years) were interviewed by using a standardized questionnaire. We found that in addition to vaccination, nonpharmaceutical interventions appeared to be protective.

Influenza A(H1N1)pdm09 virus first emerged in Mexico and southern California, USA, in early April 2009 and rapidly spread worldwide (*1*). The mode of transmission of this novel virus was similar to that of other influenza viruses. Notably, the virus disproportionately affected children and young adults (*2*). Therefore, further research was required to understand etiologic factors associated with spread of influenza A(H1N1)pdm09 among school-age children to limit transmission within schools and in the community. We conducted a case–control study to identify risk factors associated with influenza A(H1N1)pdm09 among students in Beijing, China.

## The Study

Beijing is one of the largest cities in China and has 18 districts and a population of >20 million persons. Although there is considerable variation in district size and a greater population density in urban areas, health care is accessible for residents in all districts. During the pandemic period, the Notifiable Disease Surveillance System (NDSS) was established in Beijing. Fifty-five collaborating laboratories covering all hospitals were authorized to conduct confirmation testing for influenza A(H1N1)pdm09 virus (*3*). All confirmed cases were reported through the NDSS.

Case-patients were students for whom diagnosis was confirmed during October 1, 2009–January 31, 2010. Stratified sampling was used to recruit case-patients through the NDSS. We randomly selected 3 urban and 3 rural districts from the 18 districts and listed all case-patients <18 years of age. We aimed to randomly select 50 patients from each district.

Controls were matched with case-patients at a ratio of 2:1 by sex and age (± 1 year) and were recruited from the same school and grade but from different parallel classes than case-patients. Students who reported having influenza-like symptoms since September 2009 were excluded.

The survey was conducted as a face-to-face interview by trained investigators from the Centers for Disease Control and Prevention in Beijing by using a standardized questionnaire. This interview had a 100% response rate and no data were missing. All variables were self-reported.

Data entry and statistical analysis were conducted by using EpiData software version 3.1 (www.epidata.dk/download.php) and SPSS version 16.0 (IBM, Armank, NY, USA). Bivariate and multivariate conditional logistic regression analyses were used to determine risk factors associated with infection. All variables with p<0.05 in bivariate analysis were included in multivariate analysis. Collinearity was evaluated for all variables in the final model. Backward conditional logistic regression was conducted by removing variables with p>0.10, and statistical significance was defined as p<0.05 (Technical Appendix).

A total of 304 case-patients and 608 controls were recruited from either primary or middle schools. Age range was 6–18 years for case-patients and 6–19 years for controls (median age 13 years for both groups).

Bivariate analysis identified factors associated with having influenza A(H1N1)pdm09. These factors were vaccination history, eye rubbing, handwashing immediately after sneezing, handwashing after lessons in communal classrooms, sleep time per day, participation in outdoor activities after class, population density of classrooms, classroom ventilation, mode of transportation to and from school, and participation in clustered social activities after school (Table 1).

**Table 1:** Bivariate analysis of potential factors associated with influenza A(H1N1)pdm09 infection among students ≤18 years, Beijing, China*

Variable	No. (%) case-patients, n = 304	No. (%) controls, n = 608	p value	OR (95%CI)
Vaccination against influenza A(H1N1)pdm09				
No	276 (90.8)	264 (43.4)	Referent	
Yes	28 (9.2)	344 (56.6)	<0.001	0.08 (0.05–0.12)
Vaccination with pneumococcal vaccine				
No	279 (91.8)	542 (89.1)	Referent	
Yes	25 (8.2)	66 (10.9)	0.193	0.72 (0.43–1.18)
Use of traditional Chinese medicine				
No	103 (33.9)	175 (28.8)	Referent	
Yes	201 (66.1)	433 (71.2)	0.068	0.73 (0.51–1.02)
Eye rubbing				
No	163 (53.6)	395 (65.0)	Referent	
Yes	141 (46.4)	213 (35.0)	0.001	1.68 (1.25–2.26)
Handwashing immediately after sneezing				
No	151 (49.7)	205 (33.7)	Referent	
Yes	153 (50.3)	403 (66.3)	<0.001	0.48 (0.36–0.65)
Use of soap during handwashing				
No	37 (12.2)	63 (10.4)	Referent	
Yes	267 (87.8)	545 (89.6)	0.402	0.83 (0.53–1.29)
Handwashing after lessons in communal classrooms				
No	176 (57.9)	285 (46.9)	Referent	
Yes	128 (42.1)	323 (53.1)	0.002	0.63 (0.48–0.84)
Handwashing after participation in outdoor sports activities				
No	46 (15.1)	69 (11.3)	Referent	
Yes	258 (84.9)	539 (88.7)	0.088	0.69 (0.45–1.06)
Duration of handwashing, s				
<20	176 (57.9)	347 (57.1)	Referent	
≥20	128 (42.1)	261 (42.9)	0.800	0.96 (0.71–1.30)
Sleep time, h/day				
<7	99 (32.6)	162 (26.6)	Referent	
≥7	205 (67.4)	446 (73.4)	0.030	0.67 (0.47–0.96)
Sharing of tableware with classmates				
No	263 (86.5)	534 (87.8)	Referent	
Yes	41 (13.5)	74 (12.2)	0.556	1.14 (0.74–1.75)
Classroom space/student, m^2^				
<1.6	223 (73.4)	412 (67.8)	Referent	
≥1.6	81 (26.6)	196 (32.2)	<0.001	0.17 (0.07–0.41)
Participation in outdoor activities after class				
No	232 (76.3)	411 (67.6)	Referent	
Yes	72 (23.7)	197 (32.4)	0.003	0.58 (0.40–0.83)
Frequency of classroom ventilation				
>1×/h	109 (35.9)	160 (26.3)	Referent	
1×/h	195 (64.1)	448 (73.9)	0.002	0.61 (0.44–0.83)
Having meals in small restaurants near school				
No	232 (76.3)	460 (75.7)	Referent	
Yes	72 (23.7)	148 (24.3)	0.808	0.96 (0.67–1.37)
Modes of transportation to and from school				
Closed (taxi, public transportation, school bus, car)	188 (61.8)	325 (53.5)	Referent	
Open (walking, bicycle, motorcycle)	116 (38.2)	283 (46.5)	0.009	0.66 (0.48–0.90)
Participation in clustered social activities after school closure				
No	266 (87.5)	559 (91.9)	Referent	
Yes	38 (12.5)	49 (8.1)	0.023	1.76 (1.08–2.86)

*Bivariate conditional logistic regression was used to generate p values. OR, odds ratio.

Multivariate analysis showed that in addition to vaccination, a series of environmental and behavioral factors were associated with reducing the risk for influenza A(H1N1)pdm09. These factors included provision of classroom space >1.6 m^2^/student, participation in outdoor activities after school, decreased interval of classroom ventilation, immediate handwashing after sneezing, having more sleep time (>7 h/day), and use of open modes of travel (walking, bicycle, and motorcycle) (Table 2).

**Table 2:** Multivariate analysis of independent factors associated with influenza A(H1N1)pdm09 infection among students <18 years of age, Beijing, China*

Variable	p value	Matched OR (95% CI)
Vaccination against influenza A(H1N1)pdm09		
No	Referent	
Yes	<0.001	0.07 (0.04–0.11)
Handwashing immediately after sneezing		
No	Referent	
Yes	<0.001	0.49 (0.33–0.72)
Sleep time, h/day		
<7	Referent	
>7	0.042	0.62 (0.38–0.98)
Classroom space/student, m^2^		
<1.6	Referent	
≥1.6	<0.001	0.11 (0.04–0.31)
Participation in outdoor activities after class		
No	Referent	
Yes	0.029	0.60 (0.38–0.95)
Frequency of classroom ventilation		
>1×/h	Referent	
1×/h	0.023	0.60 (0.39–0.93)
Mode of transportation to and from school		
Closed (taxi, public transportation, school bus, car)	Referent	
Open (walking, bicycle, motorcycle)	0.010	0.58 (0.39–0.88)
Participation in clustered social activities after school		
No	Referent	
Yes	0.025	2.08 (1.10–3.95)

*Ten variables were included in multivariate conditional logistic regression analysis. Backward conditional logistic regression was conducted by removing variables with p>0.10, and 8 variables remained in the final regression model. All statistical tests were 2-sided, and significance was defined as p<0.05. The statistic for each variable was obtained after adjustment for other 7 variables in the final regression model.

## Conclusions

We found several variables that determined whether students would have influenza A(H1N1)pdm09. These factors were vaccination, classroom space, outdoor activities, classroom ventilation, handwashing, sleep time, and modes of travel.

Vaccination against influenza A(H1N1)pdm09 was more common among controls than case-patients, suggesting its potential value of protection. However, the vaccination rate is low in Beijing, China. Limited knowledge and misconceptions regarding vaccination safety were contributing risk factors (*4*–*6*).

Because transmission modes for this virus appeared to be similar to those for seasonal influenza viruses, involving close, unprotected contact with respiratory droplets (*7*), we found that environmental issues appeared to be protective. When the interval of classroom ventilation exceeded 1 h, air renewal was determined to be inadequate, increasing potential risk for infection. These findings are consistent with those of other studies, which reported that influenza can spread in a confined space with insufficient air flow and that clustering of students within classrooms or during after-school activities can facilitate transmission of infectious diseases (*8*,*9*).

Social distancing might be another protective nonpharmaceutical measure. When available classroom space per student was <1.6 m^2^, there was a greater chance that students having influenza A(H1N1)pdm09 would have close contact with healthy classmates, who would be at higher risk of acquiring this disease.

We found that use of closed modes of transportation was also a risk factor. Although other studies reported that transmission rates of influenza A(H1N1)pdm09 were not increased by close and frequent contact with other persons on public transportation, we advocate use of open modes of transportation for travel to and from school, and self-protection measures when using closed modes of transportation (*10*). For instance, because wearing of face masks is easily applicable and has been shown to be protective, it tended to be a preventative measure for students who use closed transport systems (*11*).

School closure has been identified as a protective measure for controlling influenza pandemics (*12*). Some students after school closure continued to participate in clustered social activities, thereby having potentially increased their risk for contact with patients with influenza A(H1N1)pdm09 outside the school environment. Thus, after school closure, avoidance of large gatherings and clustered social activities may further reduce infection among students.

Some variables that we analyzed were not risk factors (vaccination with pneumococcal vaccine, drug prophylaxis [using traditional Chinese medicine], some handwashing habits (e.g., duration of handwashing), and sharing of tableware with classmates. Further studies might be needed to determine their effects.

There were limitations to this study. Case-patients were recruited into the study 3–8 months after receiving a confirmed diagnosis. Therefore, data collection was retrospective and had potential recall bias. Not all risk factors for influenza could be comprehensively assessed by the questionnaire. Controls were not subjected to laboratory testing, and some asymptomatic infected students may have been misclassified as controls, resulting in underestimation of odd ratios of certain risk factors and overestimation of odd ratios of certain protective factors. We did not include face mask use in the analysis because it was difficult to accurately categorize wearing face masks, given the large variety of face masks in different sizes and varying tightness in use during the pandemic in Beijing, and because we had no data for time, place, or duration of face mask use.

In conclusion, administration of vaccine and nonpharmaceutical interventions were beneficial for control of influenza A(H1N1)pdm09. Thus, it is essential to increase awareness regarding severity of influenza A(H1N1)pdm09 to improve knowledge of the protective effect of influenza vaccine and to promote use of nonpharmaceutical interventions among school-age children.

Technical Appendix Methods used to determine risk factors for influenza A(H1N1)pdm09 among students, Beijing, China.
