# Supplementary material for: Risk Factors for Influenza A(H1N1)pdm09 among Students, Beijing, China
Source: Emerg Infect Dis. 2013 Feb;19(2):309–12. doi: 10.3201/eid1902.120628 (PMC3559042; doi:10.3201/eid1902.120628)
Supplement: Technical Appendix — Methods used to determine risk factors for influenza A(H1N1)pdm09 among students, Beijing, China. [file 12-0628-Techapp_s1.pdf]

Article DOI: http://dx.doi.org/10.3201/eid1902.120628

Risk Factors for Influenza A(H1N1)pdm09 among Students, Beijing, China

Technical Appendix

Methods used to determine risk factors for influenza A(H1N1)pdm09 among students, Beijing, China

Case-Patients

During the pandemic period, a surveillance system, the Notifiable Disease Surveillance System (NDSS), was established in Beijing. Fifty-five collaborating laboratories covering all hospitals in Beijing conducted laboratory confirmation testing for influenza A(H1N1)pdm09 by real-time reverse transcription PCR. Case-patients were defined as persons with demonstrable influenza-like symptoms and laboratory evidence of influenza A(H1N1)pdm09 virus infection. All confirmed cases were reported through NDSS so that influenza A(H1N1)pdm09 could be monitored.

In this study, cases were in school-age children with a confirmed diagnosis of influenza A(H1N1)pdm09 made during October 1, 2009–January 31, 2010. Stratified sampling was used to recruit case-patients through NDSS. We randomly selected 3 urban districts and 3 rural districts from 18 districts in Beijing, and listed all patients ≤18 years of age by district. We sought to randomly select 50 patients with confirmed influenza A(H1N1)pdm09 from each district. A total of 304 case-patients were enrolled in the study, all of whom were students who attended either primary schools or middle schools (Technical Appendix Table).

Controls

Controls were matched at a ratio of 2:1 with case-patients by sex and age (± 1 year). Controls were randomly selected from the same school attended by case-patients (Technical Appendix Table). Basic demographic information such as age, sex, grade, and class was collected from the school records for each case-patient. Recruitment of eligible controls was conducted in parallel classes, but not from the same class as any of the case-patients. This measure enabled independent investigation of potential risk factors for infection, such as frequency of classroom ventilation and classroom space, for each of the case-patients and controls. A confirmed or suspected diagnosis of influenza A(H1N1)pdm09 prevented inclusion of students in the control group. Any students who reported influenza-like symptoms (including fever >37.2°C, cough, sore throat, nasal congestion, and rhinorrhea) after September 2009 were also excluded. By following these criteria, we excluded patients with possible undetected infection with influenza A(H1N1)pdm09 from the control group, thereby reducing potential bias.

Data Collection

All participants were recruited during March 2010–May 2010. The median time for recruiting case-patients was 15 weeks after illness, and the median time for recruiting controls for each case-patient was within 1 week after case-patients were recruited. This study was approved by the institutional review board and human research ethics committee of the Beijing Center for Disease Control and Prevention. Informed consent was obtained from each participant or guardian where appropriate.

Data from case-patients and controls was collected by face-to-face interviews that used a standardized questionnaire. Each participant was interviewed by 2 investigators simultaneously. One investigator was allocated the task of interviewing the student and recording answers, and the other investigator was responsible for ensuring that the questionnaire was completed. Each questionnaire was co-signed by both interviewers upon completion of the interview. Information

was collected on potential risk factors related to personal hygiene habits, vaccination history, and means of transportation. There were 19 self-reported variables: 1) classroom space per student (adequate $\geq 1.6m^2$, inadequate $<1.6m^2$); 2) participation in outdoor activities after class; 3) frequency of classroom ventilation (frequent, more than once per hour; infrequent, once per hour); 4) eye rubbing; 5) handwashing immediately after sneezing; 6) use of soap during handwashing; 7) using running water to wash hands; 8) handwashing after lessons in communal classrooms; 9) handwashing after outdoor sports activities; 10) duration of handwashing (<20 s or ≥20 s); 11) sleep time per day (more sleep time ≥7 h, less sleep time, <7 h); 12) sharing tableware with classmates; 13) having meals in small restaurants (catering for <100 persons dining simultaneously) near the school; 14) vaccination against influenza A(H1N1)pdm09; 15) vaccination with pneumovax; 16) vaccination against seasonal influenza; 17) drug prophylaxis (using traditional Chinese medicine); 18) modes of transportation to and from school (enclosed transportation such as taxis, public transportation, private cars, or school buses; open transportation such as walking, bicycles, or motorcycles); and 19) participation in clustered, social activities after school closure (such as attendance at parties).

Because the variable handwashing with soap may also include handwashing with running water, we exclude the variable hand washing with running water before analysis. We also excluded the variable vaccination against seasonal influenza because it corresponded strongly with vaccination against influenza A(H1N1)pdm09. A total of 17 variables were used in the final analysis.

All participants were interviewed independently of each other. Data were checked to ensure quality, completeness, and validity by trained staff at the Center for Disease Control and Prevention.

Statistical Analyses

Data from each questionnaire for case-patients and controls were entered in duplicate and were verified by using EpiData software version 3.1. (www.epidata.dk/download.php). Statistical analysis was performed using SPSS version 16.0 (SPSS Inc., Chicago, IL, USA). Median and range values were calculated for continuous variables, and percentages were calculated for categorical variables. Bivariate and multivariate conditional logistic regression analysis was conducted to determine risk factors associated with infection among students. Matched odds ratios and 95% CIs were calculated. Variables with $p<0.05$ by bivariate analysis were included in multivariate analysis. Collinearity was evaluated for all variables in the final model. Backward conditional logistic regression was conducted by removing variables with $p>0.10$. All statistical tests were 2-sided, and significance was defined as $p<0.05$.

Technical Appendix Table. Characteristics of students analyzed for risk factors for influenza A(H1N1)pdm09, by district, Beijing, China

	District					
Characteristic	Dongcheng	Xicheng	Haidian	Tongzhou	Changping	Miyun
Participant						
Case-patient	54	50	50	50	50	50
Control	108	100	100	100	100	100
No. schools	3	6	10	1	2	6
Median age, y (range)	12 (8–15)	13 (9–18)	10 (7–15)	14 (12–16)	11 (6–16)	15 (11–19)
Sex						
M	81	78	87	69	65	103
F	81	72	63	81	85	47
